# Timeline of hypoglossal motor neuron death and intrinsic tongue muscle denervation in high-copy number SOD1^G93A^ mice

**DOI:** 10.3389/fneur.2024.1422943

**Published:** 2024-07-25

**Authors:** Matthew J. Fogarty, Joy R. Drieberg-Thompson, Mark C. Bellingham, Peter G. Noakes

**Affiliations:** ^1^Department of Physiology and Biomedical Engineering, Mayo Clinic, Rochester, MN, United States; ^2^School of Biomedical Sciences, St Lucia, QLD, Australia; ^3^Queensland Brain Institute, St Lucia, QLD, Australia

**Keywords:** amyotrophic lateral sclerosis, motor neurons, neuromuscular junctions, hypoglossal, tongue

## Abstract

In amyotrophic lateral sclerosis (ALS) *postmortem* tissue and the SOD1 mouse model at mid-disease, death of hypoglossal motor neurons (XII MNs) is evident. These XII MNs innervate the intrinsic and extrinsic tongue muscles, and despite their importance in many oral and lingual motor behaviours that are affected by ALS (e.g., swallowing, speech, and respiratory functions), little is known about the timing and extent of tongue muscle denervation. Here in the well-characterised SOD1^G93A^ (high-copy) mouse model, we evaluated XII MN numbers and intrinsic tongue muscle innervation using standard histopathological approaches, which included stereological evaluation of Nissl-stained brainstem, and the presynaptic and postsynaptic evaluation of neuromuscular junctions (NMJs), using synapsin, neurofilament, and α-bungarotoxin immunolabelling, at presymptomatic, onset, mid-disease, and endstage timepoints. We found that reduction in XII MN size at onset preceded reduced XII MN survival, while the denervation of tongue muscle did not appear until the endstage. Our study suggests that denervation-induced weakness may not be the most pertinent feature of orolingual deficits in ALS. Efforts to preserve oral and respiratory functions of XII MNs are incredibly important if we are to influence patient outcomes.

## Introduction

Death of corticospinal neurons and the motor neurons (MNs) that innervate skeletal muscle is the pathognomic feature of amyotrophic lateral sclerosis (ALS) (1, 2). In ALS, disruption of the neuromotor system and muscle denervation leads to weakness and eventual death by respiratory insufficiency within 3 years of diagnosis (3, 4). Hence, respiratory neuromotor impairments have a strong association with disease morbidity and mortality, with ALS having effects on ventilatory (breathing) and non-ventilatory (cough and sneeze) respiratory behaviours, as well as orolingual and aerodigestive behaviours including speech and swallowing (3, 5). All of these behaviours involve some aspects of tongue musculature (6–8). These deficits (including insufficient cough and dysphagia) result in an increased risk of airway obstruction and aspiration pneumonia (4).

In patients, hypoglossal (XII) MN loss is apparent in human postmortems (2). In the most widely used murine model of ALS, hSOD1^G93A^ (SOD1) hypoglossal MN death is observed at postnatal (P) day 90 (early-disease) and endstage (~P140) in SOD1 mice (9) and rats (10). Across multiple avenues of investigation, fast fatigueable (type FF) motor units, comprising larger MNs and higher force-producing type IIx/IIb muscle fibres they innervate are vulnerable to ALS (11–14). By contrast, slow and fast fatigue-resistant (type S and FR units, respectively) motor units, comprising smaller MNs innervating low-force producing type I and IIa muscle fibres, are relatively resilient. These findings are consistent with the survival of smaller XII MNs in *postmortem* patients (2) and murine brainstem (9). The pattern of FF-type MN death and muscle fibre denervation underpins the specific behavioural vulnerability of higher-force requiring behaviours (e.g., cough) observed in ALS (4).

At presymptomatic ages [prior to MN death (9)], XII MNs of SOD1 mice have altered intrinsic and extrinsic excitability (15) and dendritic abnormalities (16) that were exclusive to larger XII MNs (17). However, it is unknown whether this early pathophysiology is matched by presymptomatic changes in XII MN survival or tongue muscle innervation, as evident in *Tibialis anterior* motor units in these SOD1^G93A^ mice (18). In this study, we assessed XII MN survival and intrinsic tongue muscle innervation at presymptomatic, onset, mid-disease, and endstage. We hypothesise that there will be a loss of larger XII MN concomitant with tongue NMJ denervations at disease onset in SOD1^G93A^ mice.

## Methods

### Ethics approval and experimental animals

All procedures were performed in accordance with national and international guidelines and were approved by the University of Queensland Animal Ethics Committee. Female and male age and litter-matched wild-type (WT; *n* = 25 for the fixed brainstem histology and *n* = 16 for frozen tongue evaluations) and high-copy number heterozygote hSOD1^G93A^ (SOD1; *n* = 24 for the fixed brainstem histology and *n* = 16 for frozen tongue evaluations) were used at four pre-defined disease stages; presymptomatic (P30), onset (P70), mid-disease (P100–120), and endstage (P130–150) (16, 19). Endstage and humane endpoints for euthanasia were determined by a paralysis of the hindlimbs and the lack of righting reflex. Animals were obtained from our female WT x male SOD1 heterozygote breeding scheme, with pups genotyped and then randomly assigned to each experimental group. Animals were maintained in filtered cages under a 12:12-h light–dark cycle with *ad libitum* access to chow and water. In all experiments, mice were deeply anaesthetised with 60–80 mg/kg of pentobarbitone (Vetcare), with levels verified by the loss of palpebral and pain reflexes.

### XII MN histology

Following deep anaesthesia, mice were intracardially perfused with phosphate-buffered saline pH 7.4 (PBS, 0.1 M) and then 4% paraformaldehyde in PBS. The brainstem was removed, sunk in 30% sucrose, subsequently frozen in liquid nitrogen, sectioned into 16 μm serial transverse cryosections, stained with 0.1% thionin (v/v in an acetic acid buffer), and imaged using a Zeiss Axioskop II microscope. XII MNs were quantified bilaterally with counts of every 5th section (20–22) with anatomical regions located with the aid of a brain atlas (23) and the 4th ventricle and central canal used as key landmarks. To estimate the XII MN volumes, the *x*, *y*, and *z* diameter of every 10th XII MN was provided for the volume of an ellipsoid.

### NMJ assessment

Following deep anaesthesia, the tongue blade was removed, and 40 μm transverse vibratome sections were cut and stained for presynaptic and postsynaptic elements of the NMJ (24). Acetylcholine receptors (AChRs) were located using Alexa-555 conjugated α-bungarotoxin [BTX; diluted 1:500 in PBS; Sigma MO, USA], and motor nerve endings were located with rabbit anti-SV2 1 [1:50, Sigma] and anti-neurofilament [1:200, Sigma], with secondary Alexa-488 goat anti-rabbit [1:500, Invitrogen] as previously described (25). All antibodies were diluted in a blocking buffer. Intrinsic tongue muscles were imaged using a Zeiss LSM 900 confocal microscope using Airyscan detectors in multiplex 4y mode using a 63x oil objective N.A. 1.4. Approximately 25 tongue NMJs were imaged per mouse, across the proximal middle and distal regions of the tongue blade. The percentage overlap between presynaptic synapsin-NF and BTX-labelled postsynaptic AChRs was used to classify innervated, partially, or fully denervated muscle fibres (18).

### Statistical analysis

Prism 9 was used for all analyses (GraphPad, Carlsbad, CA), which were performed blind to genotype. For all comparisons, two-way ANOVAs with Bonferroni *post-hoc* tests were performed, with statistical significance established at a *p*-value of <0.05. All data were reported as the mean ± 95% CI of the mean.

## Results

### Loss of bodyweight by endstage in SOD1 mice

Both female and male mice gained weight until mid-disease, where endstage SOD1 mice (19.3 ± 1.2 g, *n* = 13) weighed ~12% less than WT controls (22.1 ± 1.3 g, *n* = 13; *p* = 0.0123, unpaired t-test).

### Loss of larger XII MNs in SOD1 mice

**Figure 1:**
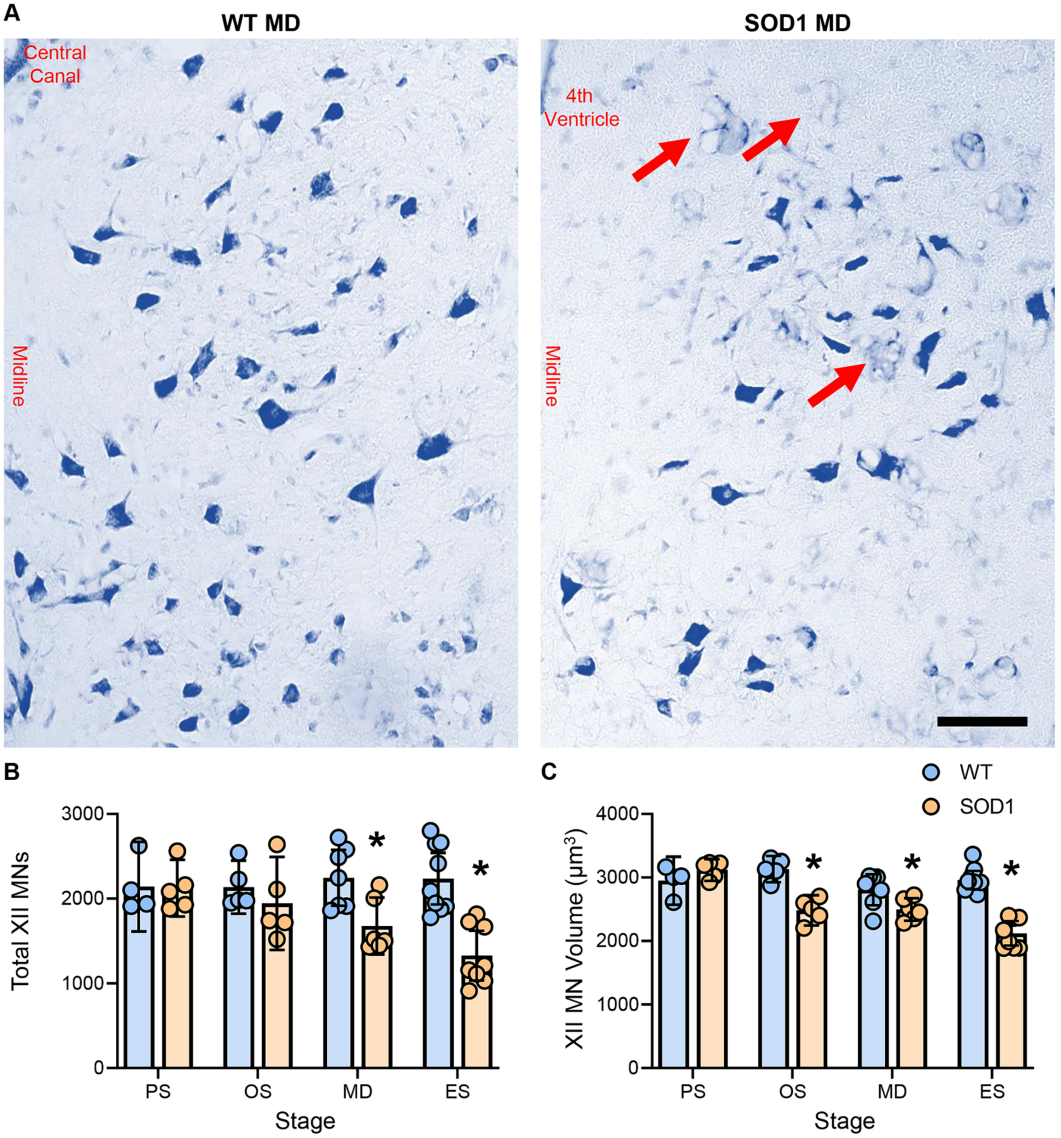
**(A)** Photomicrographs of the Nissl-stained brainstem and XII MNs in WT and SOD1 mice at mid-disease (MD). **(B)** Plot showing a reduced number of XII MNs in SOD1 mice at mid-disease and endstage (ES) but not presymptomatic (PS) or onset (OS). Note that the XII MN number is relatively stable until mid-disease, where ~25% of XII MNs have perished. Red arrows indicate vacuolated regions within the hypoglossal nucleus from mid-disease in SOD1 mice. **(C)** Scatterplot showing reduced XII MN volume in SOD1 mice from the onset. These somal changes are not the earliest alterations in XII MN morphology, but they are consistent with the resilience of type S and FR motor units. Two-way ANOVAs with Bonferroni post-tests, * indicates *p* < 0.05 between genotypes within an age group. Each symbol represents one mouse (i.e., the *n*). Scale bar = 100 μm.

XII MN numbers were evaluated bilaterally in the entire XII nucleus in WT and SOD1 mice (Figure 1). XII MN loss was dependent on genotype (*F*_(1.41)_ = 16.5, *p* = 0.0002) and genotype–age interactions (*F*_(3.41)_ = 4.0, *p* = 0.014; two-way ANOVA), with significant reductions (~25%) at mid-disease (WT: 2247 ± 330, *n* = 7; SOD1: 1678 ± 336, *n* = 6; *p* = 0.023) and endstage (~41%; WT: 2236 ± 304, *n* = 9; SOD1: 1328 ± 294, *n* = 8; *p* < 0.0001) in SOD1 mice (Bonferroni post-tests; Figure 1B). No differences in XII MN number were apparent at presymptomatic (WT: 2144 ± 429, *n* = 4; SOD1: 2123 ± 336, *n* = 5; *p* > 0.99) or onset ages (WT: 2136 ± 314, *n* = 5; SOD1: 1945 ± 550, *n* = 5; *p* > 0.99; Bonferroni post-tests; Figure 1B). In SOD1 mice at mid-disease stages, extensive vacuolation of the hypoglossal nucleus was apparent, similar to that previously observed at endstage (26).

XII MN volume was dependent on genotype (*F*_(1.41)_ = 45.0, *p* < 0.0001) and genotype–age interactions (*F*_(3.41)_ = 12.4, *p* < 0.0001; two-way ANOVA), with significant reductions at onset (~21%; WT: 3132 ± 207 μm^2^, *n* = 5; SOD1: 2484 ± 238 μm^2^, *n* = 5; *p* < 0.0001), mid-disease (~11%; WT: 2808 ± 244 μm^2^, *n* = 7; SOD1: 2498 ± 178 μm^2^, *n* = 6; *p* = 0.0386), and endstage (~28%; WT: 2960 ± 145 μm^2^, *n* = 9; SOD1: 2121 ± 194 μm^2^, *n* = 8; *p* < 0.0001) in SOD1 mice (Bonferroni post-tests; Figure 1C). No difference in XII MN volume was apparent in presymptomatic mice (WT: 2950 ± 389 μm^2^, *n* = 4; SOD1: 3120 ± 171 μm^2^, *n* = 5; *p* > 0.99; Bonferroni post-test; Figure 1C).

### Tongue NMJ denervation occurs subsequent to XII MN loss in SOD1 mice

**Figure 2:**
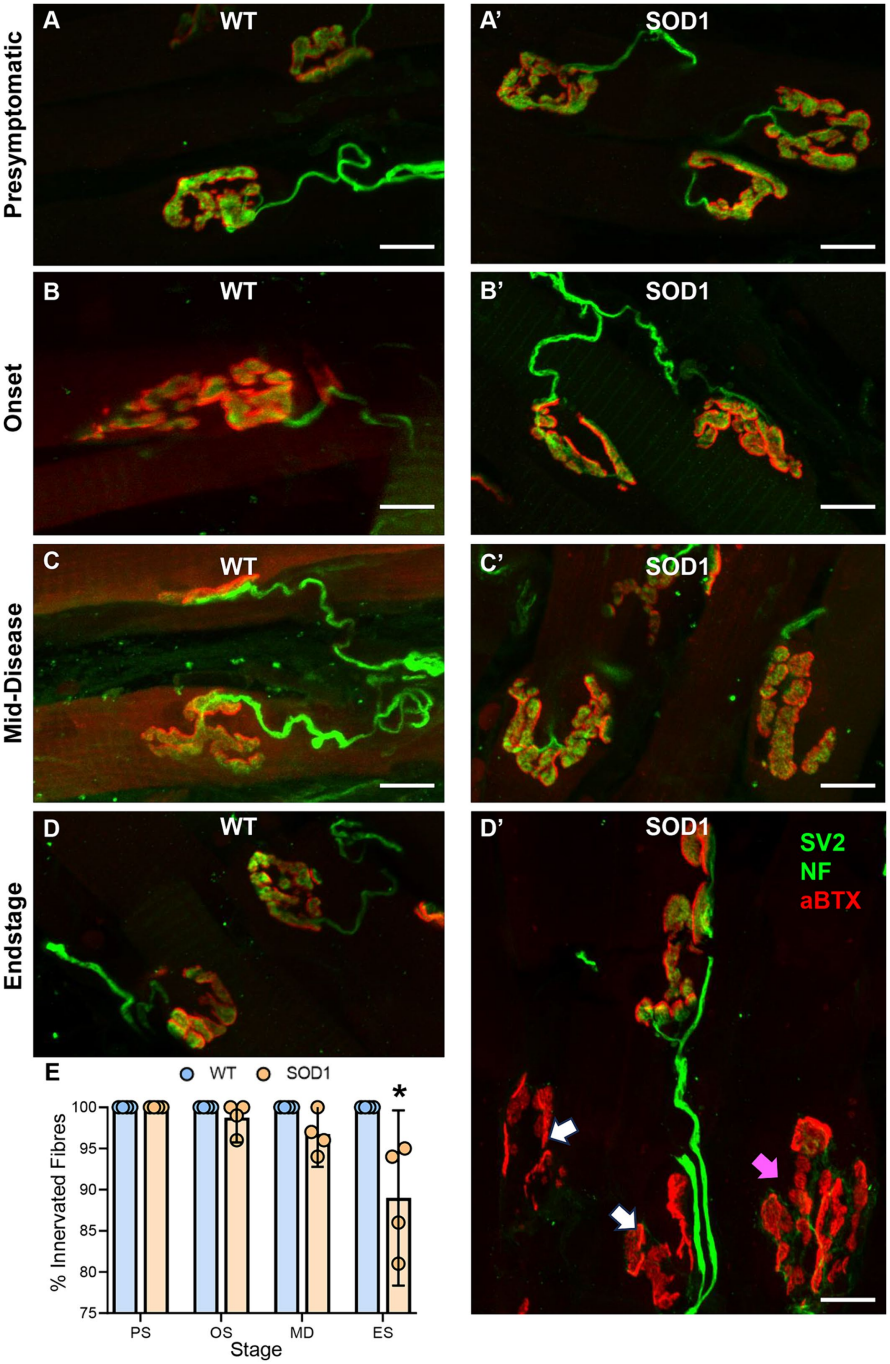
**(A–D’)** Show flattened *z*-stack projections of NMJs from wild-type (WT, *left column* **A–D**) to aged match SOD1^G93A^ (SOD1, *right column*, **A’–D’**), at all stages of ALS-like disease progression, including, presymptomatic (PS), onset (OS), mid-disease (MD), and end-stage (ES). In panel **(D’)**, white arrows indicate denervated endplates at ES, and purple arrows indicate partially denervated endplates. In all panels, presynaptic components of the NMJ (green) are labelled with antibodies to SV2 and neurofilament (NF), while postsynaptic acetylcholine receptors (red) are located with αBTX. **(E)** Scatterplot shows a reduction in the percentage (%) of innervated muscle fibres in SOD1^G93A^ mice at endstage, following XII MN loss, with innervation unchanged at presymptomatic, onset, and mid-disease. Two-way ANOVA with Bonferroni post-tests. * indicates *p* < 0.05 between genotypes within an age group. Each symbol represents one animal (i.e., the *n*) with data based on ~25 NMJs per tongue. Scale bars = 10 μm.

Intrinsic tongue muscle NMJs were evaluated whole-mount within 40-μm transverse cryosections of the tongue blade (Figure 2). The % of NMJ endplates that were innervated was dependent on genotype (*F*_(1.24)_ = 17.6, *p* = 0.0003) and genotype–age interactions (*F*_(3.24)_ = 7.1, *p* < 0.0014; two-way ANOVA). We did not observe any denervated fibres in WT mice, and denervation was rare in SOD1 mice at presymptomatic (100% innervated), onset (~99% innervated), and mid-disease (~97% innervated). However, at endstage, there was an 11% reduction of innervated fibre SOD1 mice, compared to WT (SOD1: 89 ± 10%; *p* < 0.0001, Bonferroni post-test; *n* = 4 all ages/groups; Figure 2B).

## Discussion

In the present study, we report the first longitudinal study of XII MN size-dependent survival and tongue blade innervation in SOD1 mice. This study has important implications regarding the interpretation of pathophysiological changes in young SOD1 XII MNs, and in relation to the natural history of MN death and denervation in SOD1 mice more generally. Notably, our current findings suggest that in the case of orolingual and aerodigestive behaviours, disordered neural networks altering the pattern of MN activation may be a more salient feature of dysfunction than muscle denervation and weakness *per se,* as maximal tongue strength seems maintained in SOD1 rats (27) and when corrected for baseline values in SOD1 mice (28). For behaviours such as swallowing, where multiple brainstem MN pools are activated, swallow deficits occur in SOD1 mice (26, 29) and rats (27) in the absence of reduced nucleus ambiguus MN density counts (10), although stereological assessments of MN numbers and detailed assessment of the computational capacity of these nucleus ambiguus MNs have not been comprehensively assessed [see (30)]. How aberrant brainstem neural circuitry and synaptic transmission very early in the pathogenesis of SOD1 mice (15–17) influences orolingual and aerodigestive activities at later disease stages (26, 29) remains unclear. Exploring these nuances may yield sensitive diagnostic hallmarks and widen therapeutic windows.

Our findings of XII MN loss from mid-disease are not surprising given the prior studies in P90 and endstage SOD1 mice (9, 26). Here, we extend that finding by showing that XII MN loss is not apparent at younger ages, despite widespread presymptomatic functional and morphological changes (15–17). In a major advance, we show that at onset, the surviving XII MNs of SOD1 mice are smaller than their WT comparators, consistent with reduced volumes at mid-disease and endstage. This observation suggests that the larger MNs, likely comprising type FF motor units, are vulnerable to loss (2, 9, 12–14). Indeed, the major differences in XII MNs compared to lumbar MNs in SOD1 mice seem to be their capacity to compensate for their altered synaptic milieu which is evident in neonates (15).

In XII MNs, morphological changes consistent with lowered intrinsic MN activity (i.e., dendritic expansion) are present presymptomatically in the larger more vulnerable XII MNs (16, 17). By contrast, in the more rapidly degenerating lumbar MNs and corticospinal neurons, neuronal death is apparent presymptomatically or at onset (18, 31). Notably, corticospinal neurons and lumbar MNs do not exhibit neuroplastic changes consistent with compensation but display degenerative morphologies (16, 32, 33). This compensation is not related to the respiratory neuromotor system in general, as there are marked deficits in respiratory MNs and muscles in SOD1 models (34), including the tongue (35).

Despite evidence of XII MN loss (2, 9), degeneration (26), and a plethora of early pathophysiological changes (15–17), the integrity of tongue NMJs has not been evaluated in ALS. Here, we show that gross NMJ morphology is relatively well preserved in SOD1 mice, with pathology largely absent until endstage, similar to SOD1 rats (10, 27). Importantly, our NMJ assessments do not discriminate the dynamic re-innervation of tongue muscle fibres, where formerly denervated fibres are innervated by nascent axonal projections from surviving XII MNs or fibres that have been totally unaffected. This is a key caveat to our interpretations as it has been shown that dynamic structural and functional NMJ remodelling precedes MN death in the limb muscles of SOD1^G93A^ mice (36, 37). Perhaps most relevant to ALS are recent observations in muscle from early staged ALS patients who show type 1 fibre type grouping is evident during the onset of leg muscle weakness in ALS patients, supporting the idea of re-innervation by resilient type S MNs (11). These observations, along with the inclusion of some type I and IIa fibres in the mouse tongue (38), may confound our NMJ evaluations. Regardless, the timing of pathology, including XII MN loss occurring just prior to NMJ denervation, suggests that the complete denervation of NMJs may be a consequence, not a cause of MN deficits in SOD1^G93A^ mice, consistent with neonatal XII MN dysfunction in this ALS model (15).

In the respiratory neuromotor system, expulsive behaviours, such as cough and sneeze, requiring the activation of type FF motor units, are impaired in ALS patients (4), consistent with ALS pathology selectively afflicting larger MNs (2). For the tongue muscles, ballistic-type activations occur during swallowing, which is impaired in ALS (3). There are few ways to directly assess the maximal activations of individual intrinsic and extrinsic tongue muscles. Reduced uniaxial approaches (complicated by the complex interdigitation of tongue musculature) give unequivocal maximum specific forces at the cost of being *ex vivo*, while *in vivo* nerve stimulation may be confounded by NMJ or axonal pathology (6). Alternatively, behavioural tasks, such as licking and swallowing, altered in low-copy SOD1^G93A^ mice (29, 39), can be evaluated, although interpretations are couched, as the involvement of other muscle groups is required for these activities (particularly the nucleus ambiguus for swallowing). Regardless, these tongue deficiencies are also a likely cause of the poor grooming and coat conditions of SOD1 mice from both strains.

As ALS is an age-associated neurodegenerative condition, separating the ravages of age from the effects of the disease, particularly in animal models, is often overlooked. In rodents (20, 40–43) and humans (44, 45), ageing-related XII MN loss, tongue weakness, and impaired orolingual and aerodigestive behaviours are readily observed. An important goal is to uncover the precise pathophysiological phenomena differentiating ALS from old age sarcopenia (*cf* old age vigour, or healthy ageing). To this end, the low-copy number SOD1^G93A^ mice have been employed, with their onset of symptoms is delayed compared to the high-copy number strain used in the present study (46). Although swallowing and licking behavioural deficits are identified (29, 39) and smaller genioglossal muscle fibre cross-sectional areas are evident at endstage (39), corticospinal involvement has not been clarified [see (47)], and thus, the validity of this strain is unverified. Notably, the endstage in the low-copy strain occurs at ~240 days (29, 46), barely aged enough to be considered “young” in rodent ageing studies (48). Efforts to create animal models where the timing of abnormalities can be controlled (i.e., where MN death can commence in ages approaching higher risk in humans) are likely to be highly informative and are within our grasp (49–51).

In conclusion, we have novel results showing a relative resilience of tongue muscle to denervation in SOD1^G93A^ mice, and XII MN loss consistent with prior neural (9) and behavioural (26, 29, 52) assessments. These results are also largely in agreement with unaltered maximal licking force (27) and absent (27) to mild (10) evidence of tongue denervation in SOD1 rats. In addition, we show altered morphology of XII MNs, consistent with the vulnerability of type FF motor units occurring prior to statistically robust XII MN death and tongue denervation. Whether the relative delay in XII MN and tongue denervation compared to limb MNs and muscle denervation is related to the increased capacity of the XII MNs to effect adaptive compensatory changes, rather than early pathophysiological degenerations, is an exciting new avenue to pursue (53, 54). Therapeutic assessments designed to preserve MNs would do well to include aerodigestive behaviours, particularly of swallows evoked using naturalistic methods [see (55, 56)], as there is major brainstem sensory degeneration in SOD1 mice (26). Due to their highly relevant implications for both disease morbidity and mortality, efforts assessing the bulbar manifestations of ALS, which occur even in limb-onset patients, are of great importance.

## Data availability statement

The original contributions presented in the study are included in the article/supplementary material, further inquiries can be directed to the corresponding author.

## Ethics statement

The animal study was approved by the University of Queensland Animal Ethics Committee. The study was conducted in accordance with the local legislation and institutional requirements.

## Author contributions

MF: Conceptualization, Data curation, Formal analysis, Funding acquisition, Investigation, Methodology, Supervision, Validation, Visualization, Writing – original draft, Writing – review & editing. JD-T: Conceptualization, Data curation, Formal analysis, Investigation, Writing – original draft, Writing – review & editing. MB: Conceptualization, Data curation, Formal analysis, Funding acquisition, Investigation, Project administration, Supervision, Writing – original draft, Writing – review & editing. PN: Conceptualization, Data curation, Formal analysis, Funding acquisition, Investigation, Project administration, Supervision, Writing – original draft, Writing – review & editing.
